# Early cardiovascular and respiratory changes after etorphine immobilization and naltrexone reversal in sheep

**DOI:** 10.3389/fvets.2026.1850859

**Published:** 2026-07-13

**Authors:** Anna Binetti, Hathaipat Rattanathanya, Friederike Pohlin, Ulrike Auer, J. Peter Schramel, Marlies Dolezal, Szilvia Kalogeropoulu, Cosima L. Gösele, Lisa Stahl, Lisa J. Ebenhofer, Christina Braun, Gabrielle Stalder, Martina Mosing

**Affiliations:** 1 Anaesthesiology and Intensive Care, Clinical Centre for Small Animals, Clinical Department for Small Animals and Horses, University of Veterinary Medicine Vienna, Vienna, Austria; 2 Department of Interdisciplinary Life Sciences, Research Institute of Wildlife Ecology, University of Veterinary Medicine Vienna, Vienna, Austria; 3 Platform for Bioinformatics and Biostatistics, Department of Biological Sciences and Pathobiology, University of Veterinary Medicine Vienna, Vienna, Austria

**Keywords:** electrical impedance tomography, opioid induced pulmonary hypertension, opioids antagonist, ultra potent opioids, ungulates

## Abstract

**Introduction:**

The immediate cardiorespiratory responses to etorphine immobilization and reversal with naltrexone in wildlife are underexplored.

**Objective:**

To evaluate the early cardiorespiratory changes after etorphine immobilization and reversal with naltrexone in sheep, as a model for wild ungulates.

**Methods:**

Six sheep instrumented with electrical impedance tomography (EIT) belt, arterial and pulmonary arterial catheters were immobilized with intramuscular etorphine (0.05 mg kg^−1^) and reversed with intravenous naltrexone (1 mg kg^−1^) 26 min later. Mean pulmonary arterial pressure (MPAP) and EIT derived variables (minute tidal impedance variation-TIV_MIN_; tidal impedance variation–TIV; respiratory rate–RR; end-expiratory lung impedance-EELI) were recorded continuously from 5 min before to 10 min after each drug. Arterial blood gasses were drawn at baseline and 5 min after each drug. Statistical analysis was performed in R to determine the timing of significant deviations from baseline, defined as the mean of the 5 min preceding drug administration, and compared with each minute thereafter. Post-administration values were expressed as ratios to baseline and analyzed using two-sided one-sample *t*-tests with False Discovery Rate (FDR) correction at 10% for multiple comparisons; FDR-adjusted *p*-values (*q*-values) < 0.10 were considered significant.

**Results:**

Three minutes after etorphine, MPAP increased (*q* = 0.08, median 42%), accompanied by a decrease in TIV_MIN_ (*q* = 0.09, median 40.9%) due to decrease in TIV and RR. Arterial blood gasses confirmed hypoxemia (PaO₂ median [range]: 57.1 [26.9–93.6] mmHg) and hypercapnia (PaCO₂ 49.45 [40–61.3] mmHg). Conversely, regional ventilation distribution and EELI remained unchanged. One minute after naltrexone, median RR (*q* = 0.04) and TIV_MIN_ (*q* = 0.05) increased by 119.3 and 223.6% from baseline respectively, followed by increase in TIV from minute 4 (*q* = 0.06, median 50.8%). Arterial blood gasses improved marginally (PaO₂ 63.5 [48.6–115.7] mmHg; PaCO₂ 46 [41.5–52.4] mmHg) while MPAP did not return toward baseline.

**Conclusion:**

Etorphine immobilization caused early pulmonary hypertension followed by progressive hypoventilation without ventilation redistribution. Naltrexone rapidly restored ventilation, whereas pulmonary vascular effects persisted.

## 1 Introduction

Potent opioids, particularly etorphine, are routinely used for the chemical immobilization of large wild herbivores due to their rapid onset, reliability, and reversibility (1–3). Despite these advantages, etorphine induced immobilization is frequently complicated by severe cardiovascular and respiratory compromise, most critically manifesting as hypoventilation and hypoxemia (2–5). Tachycardia, systemic and pulmonary hypertension, commonly reported in etorphine immobilized ungulates, alter pulmonary blood flow distribution, contributing to ventilation-perfusion mismatch, impaired gas exchange and decrease in oxygen delivery (2–4, 6, 7). Collectively, these effects make the period immediately following etorphine administration one of the most physiologically unstable phases during etorphine immobilization.

Naltrexone is a potent opioid antagonist that has been shown to safely and effectively reverse both the desired and adverse effects of etorphine in various wildlife species (2, 3, 5, 8–10).

Most studies evaluating the cardiorespiratory effects of etorphine and its reversal with naltrexone are limited to the later immobilization and early recovery phases rather than the periods immediately before and after drug administration (2, 3, 6, 11). During these critical transition phases, free-ranging animals are not yet safely approachable, limiting assessment to variables that can be evaluated from a distance, such as respiratory rate or obvious behavioral responses, while detailed physiological measurements requiring instrumentation or close contact are typically not feasible. As a result, the immediate cardiorespiratory responses to etorphine administration and subsequent naltrexone reversal are poorly characterized. This limitation represents an important gap in our understanding of the early physiological effects of these agents. To address this, we used sheep as a model for wild ungulates, offering a practical and ethically acceptable surrogate due to their herbivorous physiology, ease of handling, and established use in research (5).

Thoracic electrical impedance tomography (EIT) is a non-invasive imaging technique to evaluate ventilation and its regional distribution using only an electrode belt placed around the thorax. It has been used in different animal species and can be used to assess ventilation variables without requiring spirometry, facemask application, or endotracheal intubation, making it particularly suitable for measurements in the awake animal as well as during transition from awake to immobilized state and vice versa (12–18).

The aim of this study was to provide a temporal characterization of the immediate and early cardiorespiratory responses to etorphine immobilization and subsequent reversal with naltrexone in sheep by evaluation of physiological variables in the awake state prior to etorphine administration, during the transition to immobilization, and following reversal, during recovery. We hypothesized that: (1) etorphine administration would initially increase mean arterial and mean pulmonary pressures followed by a decrease in ventilation, (2) reversal with naltrexone would return these changes to physiological ranges in the same order.

## 2 Materials and methods

### 2.1 Ethical approval

This study was approved by the Ethics and Animal Welfare Committee of the University of Veterinary Medicine Vienna and authorized by the Austrian Federal Ministry of Education, Science and Research (BMBWF 2023–0.276.137). All procedures performed during the experiments adhered to the principles of Good Scientific Practice (GLP) and strictly followed the Animal Research: Reporting of *In Vivo* Experiments (ARRIVE 2.0) guidelines.

### 2.2 Animals and husbandry

**Figure 1:**
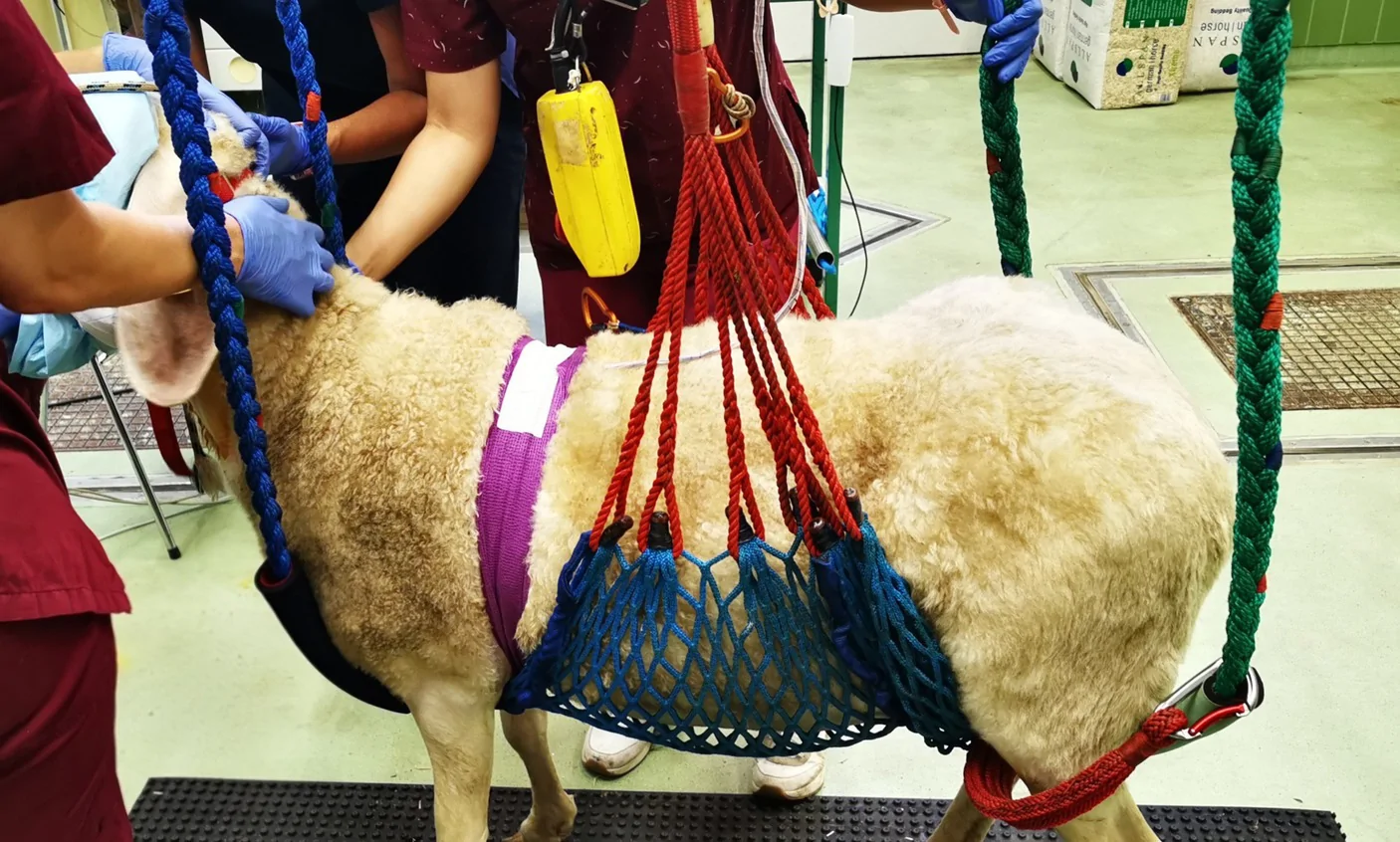
Custom-made sling used to support and maintain the sheep standing position throughout the experiment. The sling consisted of a meshed ventral support with padded ropes positioned in the axillary and inguinal regions attached to a height-adjustable crane mounted on the ceiling.

Six adult female Tirolean Mountain sheep (*Ovis aries*), with a mean body weight of 92.3 ± 8.5 kg and median age of 5.8 years (range 5.6–6.2 years) were enrolled in this study. Hematology and serum biochemistry analysis was performed 3 weeks prior to the start of this project in all animals and were all within normal limits. The sheep were housed in an outdoor enclosure of appropriate size with free access to hay and water and provision of supplemental species appropriate food concentrates once daily. To minimize handling stress during the study period, animals were habituated to manipulation including the use of a custom-made sling (TBTN®, GTRD®, Switzerland, Figure 1) over several months before the experiments.

### 2.3 Study design

This study was conducted as part of a larger prospective, randomized controlled crossover study (19). Each sheep underwent three immobilizations with intramuscular (IM) etorphine (0.05 mg kg^−1^, Captivon, Wildlife Pharmaceuticals, White River, South Africa) and two different medical interventions with one control group receiving sterile water only. Only sheep from the control group (*n* = 6) were used in the current study. The etorphine effect was antagonised with intravenous (IV) naltrexone (1 mg kg^−1^, Trexonil, Wildlife Pharmaceuticals Pty Ltd., White River, South Africa) 26 min after etorphine in all sheep. There was a 4 week washout period between immobilizations.

### 2.4 Anesthesia and instrumentation

One week before the trial, the sheep were transported to the experimental facility (Veterinary University Vienna) for acclimatisation. The day before the trial all sheep were deemed healthy based on thorough clinical examination. Both ears, the left jugular region and a 10 cm wide band around the thoracic circumference were clipped. Animals were fasted for 16 h prior to the immobilization.

On the study day, the sheep were positioned in the custom-made sling connected to a crane mounted on the ceiling. For instrumentation and prior to drug administration, animals remained standing in the sling (Figure 1).

Following placement in the sling, midazolam (0.3 mg kg^−1^, Midazolam 5%, Oberösterreichische Gesundheitsholding GmbH, Linz, Austria) was injected IV to facilitate instrumentation. A 32 electrodes custom-made single plane stretchable EIT belt was positioned around the thorax on the clipped band at the level of the 5th, 6th intercostal space. Electrode gel was generously applied to the skin to ensure stable contact between the electrodes and the skin. The belt was then connected to the EIT device (BBVet, (SenTec AG, Landquart, Switzerland) to record impedance derived ventilation parameters (47 frames, skip 4 pattern). To guarantee contact between the electrodes and the skin and to avoid drying of the electrode gel, the EIT belt was further stabilized and maintained in place using a stretchable self-adhesive bandage (Cop-cohesive colored bandage, Hohenwallner Medizin & Veterinärbedarf GmbH, Leonding, Austria) placed on top of the belt around the thoracic circumference.

After aseptic preparation and infiltration of 2 mL lidocaine subcutaneously (Xylanaest® purum 2%, Gebro Pharma GmbH, Fieberbrunn, Austria) at the level of the left mid neck region, an 8.5F percutaneous sheath introducer (Teleflex®, Arrow international LLC, Morrisville, NC, United States) was placed to allow for drug administration and facilitate the insertion of a 7.5F thermodilution Swan–Ganz catheter (Swan-Ganz™ Catheter, Edwards Life Sciences, Irvine, CA, United States). This catheter was advanced under continuous pressure waveform guidance for pulmonary arterial pressures monitoring. Concurrently, an 18G catheter (Vasofix® Safety, B. Braun Melsungen AG, Melsungen, Germany) was aseptically placed in the right auricular artery for invasive systemic arterial pressure measurement. All vascular catheters were connected to pre-calibrated pressure transducers, zeroed to atmospheric pressure and leveled at the height of the scapulohumeral joint.

Standard adhesive electrocardiogram (ECG) electrodes were positioned on shaved areas over both carpi and the sacral region for heart rate and rhythm evaluation.

All monitoring devices were interfaced with the PowerLab data acquisition system (PowerLab C, 26 series and T series, AD Instruments, Dunedin, New Zealand) for synchronous, continuous data collection at a sampling rate of 100 kS/s (100.000 samples s^−1^).

After completion of instrumentation, flumazenil 0.01 mg kg^−1^ (Flumazenil Kabi, Fresenius Kabi Austria GmbH, Graz, Austria) was administered IV to reverse the effects of midazolam. A standardized 45-min washout interval was allowed after flumazenil injection and prior to baseline (resting state) data collection.

### 2.5 Experimental procedure and data collection

**Figure 2:**
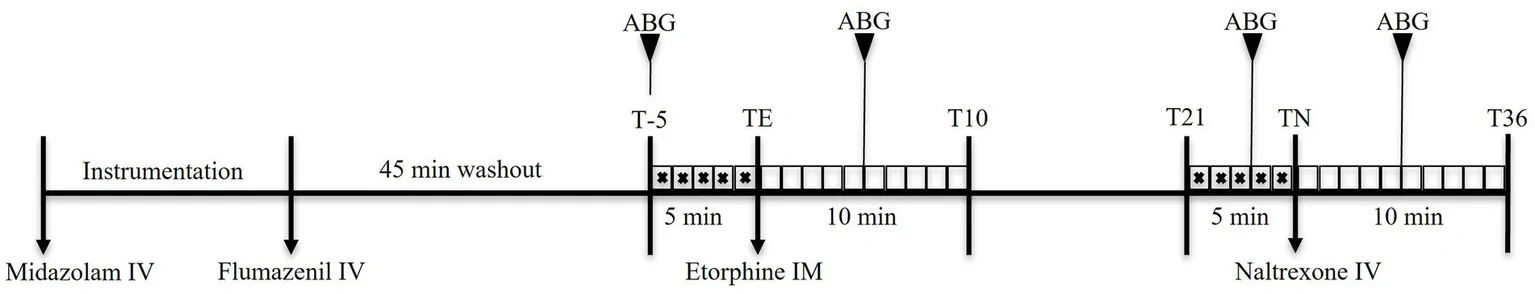
Study timeline. Sheep (*n* = 6) were instrumented following IV midazolam. After completion of instrumentation, IV flumazenil was administered and a 45-min washout period allowed. Cardiorespiratory and EIT variables were recorded continuously and analyzed in 1-min intervals, represented by individual squares (each square = 1 min; crossed: pre-administration; empty: post-administration). Measurements were obtained from 5 min before (T − 5) to 10 min after (T10) etorphine administration (TE), and from 5 min before (T21) to 10 min after (T36) naltrexone administration (TN). Arterial blood gasses (ABG) were drawn at baseline (5 min and 2 min before etorphine and naltrexone respectively) and 5 min after each drug administration.

Etorphine (0.05 mg kg^−1^) was administered IM in the quadriceps femoral muscle region (timepoint TE). Data acquisition started 5 min before and continued for 10 min after etorphine administration (Figure 2).

After etorphine injection, the sheep became immobilized and settled within the sling. The animals were maintained in a supported sternal posture with a blindfold over the eyes. The head was held upright by an assistant. The sternum rested against the sling and the limbs were suspended within it while still touching the floor. This setup mimicked a standardized sternal recumbency while ensuring safe restraint [(19), Figure 1].

Seven minutes after etorphine administration, the sheep received IV sterile water (0.08 mL kg^−1^) as a control treatment. Blood gas analyses and cardiovascular variables were collected at predefined intervals as part of the larger study protocol.

Naltrexone (1 mg kg^−1^) was injected IV 26 min after immobilization (timepoint TN) to reverse etorphine.

Again, all cardiovascular and respiratory variables as well as EIT measurements were continuously recorded from 5 min before to 10 min after TN (Figure 2).

Heart rate (HR) was recorded from ECG, mean arterial pressure (MAP) via the auricular artery catheter and mean pulmonary artery pressure (MPAP) from the distal port of the Swan-Ganz catheter. All ventilation data were retrospectively evaluated from EIT measurements.

ECG recordings were evaluated for arrhythmias. Sinus tachycardia and bradycardia were defined as a HR above 120 beats min^−1^ and below 60 beats min^−1^, respectively.

Clinical and behavioral signs of etorphine immobilization were assessed by direct observation and recorded throughout the experimental period.

Additionally, arterial blood gas samples were drawn anaerobically from the arterial catheter into a pre-heparinized syringe (BD VACUTAINER®, Becton Dickinson Company, Franklin Lakes, NJ, United States) and analyzed immediately (Cobas® b 123 POC system, Roche Diagnostics GmbH, Mannheim, Germany) at multiple predefined timepoints as part of the larger experimental protocol. In the present analysis, for evaluation of arterial partial pressure of oxygen (PaO_2_) and carbon dioxide (PaCO_2_), specific timepoints were selected: measurements obtained 5 min before etorphine and 2 min before naltrexone administration were defined as pre-administration values, while measurements obtained 5 min after each respective drug were defined as post-administration values.

Experimental termination criteria were predefined using the following criteria:

1. Mean pulmonary arterial pressure exceeded 50 mmHg or.
2. Apnoea persisting for more than 30 s.

In such cases, naltrexone (1 mg kg^−1^) was administered IV to reverse immobilization independent of the study stage. Data collected up to the point of termination were retained for analysis. After completion of recordings, all monitoring equipment was removed. At the end of each trial, all sheep received 0.5 mg kg^−1^ Meloxicam (Metacam®, Boehringer Ingelheim, Ingelheim am Rhein, Germany) IM for analgesia and were observed until full recovery before returning to their enclosure.

### 2.6 Post-hoc analysis for EIT variables

For calculation of EIT variables, 10 breaths, free of movement artefact, were analyzed. EIT data were processed using a dedicated software (IBEX® Software, SenTec AG, Therwil, Switzerland). All impedance measurements were expressed in arbitrary units (AU). To account for interindividual variability due to the use of AU for impedance changes, values were presented as ratios relative to baseline. Tidal impedance variation (TIV), defined as the difference in impedance between the beginning and end of inspiration, was calculated as a surrogate measure of tidal volume (20, 21). The impedance at the onset of inspiration, referred to as end-expiratory lung impedance (EELI), represents thoracic impedance prior to inspiration defined as a surrogate for functional residual capacity (FRC) (22, 23).

Other evaluated EIT derived variables included center of ventilation right to left (CoVRL) and ventral to dorsal (CoVVD), which describe the spatial distribution of ventilation along the horizontal and vertical thoracic axes, respectively. The region of interest of the right (ROIR) and left lung (ROIL) represent the proportion of total ventilation occurring within each hemithorax. Inspiratory time (Ti) was defined as the time interval between the onset of the inspiratory increase in thoracic impedance and the point of peak impedance within a single breath cycle, as determined from the global EIT impedance-time curve. Respiratory rate (RR) was defined as the number of complete impedance-derived breathing cycles per minute, calculated from the global EIT impedance–time curve and minute tidal impedance variation (TIV_MIN_) as a product of TIV and RR.

Evaluated EIT-derived flow variables included global peak inspiratory (PIF) and peak expiratory flow (PEF), derived from the first derivative of the respective impedance–time curves and expressed in arbitrary units per second. Regional peak inspiratory and expiratory flows were calculated separately for the right and left lung regions of interest. Peak inspiratory flow right (PIFR) and peak expiratory flow right (PEFR) were defined as the maximal inspiratory and expiratory impedance change rates within the right lung region, whereas peak inspiratory flow left (PIFL) and peak expiratory flow left (PEFL) were defined analogously for the left lung region. All EIT flow variables were normalized to TIV.

### 2.7 Statistical analysis

All statistical analyses were performed in R (v4.4.1; R Core Team, 2024). Data were prepared using functions from packages dplyr v1.1.4 (24). Figures were produced with functionality from package ggplot2 v3.5.1 (25) and exported in scalable vector graphics format via package svglite v2.1.3 (26).

For all variables, mean values were calculated for each minute spanning from 5 min before to 10 min after drug administration. To facilitate assessment of temporal changes, measured variables were normalized to their reference (baseline) before drug administration. This baseline was defined as the average of the five 1-minute mean values obtained before each drug administration, resulting in a single reference value used to calculate ratios. Therefore, all data are presented as ratios relative to baseline.

Only timepoints with a minimum of 4 observations for a given variable at a given post-administration timepoint were analyzed.

Minute 5 following etorphine and minute 2 following naltrexone were excluded from statistical analysis due to equipment failure affecting one sheep. In addition, MAP and MPAP measurements were unavailable 5 and 6 min after both etorphine and naltrexone injection in five of the six sheep due to arterial blood gas sampling and catheter line flushing. Consequently, these timepoints were excluded from statistical analysis for these variables.

After verifying that normal distribution assumptions were not violated, hypothesis testing was done using a two sided, one sample t-test (function *t-*test, with options *mu = 1* and *alternative = “two.sided”*) against a constant of one under the null hypothesis (i.e., ratio to baseline equals one if there is no change after drug administration to the baseline measure, expressed as mean of the 5 measures before administration).

*p*-values obtained from the two-sided one sample *t*-tests were adjusted for multiple comparisons using the Benjamini–Hochberg procedure to control the false discovery rate (FDR) at 10% (27), yielding FDR-adjusted *p*-values (*q*-values).

Statistical significance was defined at a global 10% FDR (*q* < 0.10) across all 10 post-administration timepoints and across all measured variables within each administered drug.

Results are presented as estimated mean ratios with their 95% confidence intervals for each timepoint. Graphically black dots represent means not significantly different from one, red dots indicate significant difference from 1 at 10% FDR. Confidence intervals are shown as gray bars. A ratio not significantly different from one indicates no change from baseline; estimates significantly >1 indicate an increase and estimates significantly <1 indicate a decrease relative to baseline.

## 3 Results

### 3.1 General results

All sheep (*n* = 6) completed the study protocol. In one sheep, naltrexone was injected earlier than scheduled (22 min after etorphine instead of 26 min) due to mean pulmonary arterial pressure exceeding the predefined termination endpoint of 50 mmHg. Data acquisition relative to naltrexone for this sheep remained the same, with 5 min of baseline and 10 min of post-administration data available for analysis. The animal recovered uneventfully.

Following etorphine administration, the first observable clinical and behavioral signs of etorphine immobilization occurred at a median of 3 min (range 2–4 min) and included excitation, stiffness, shivering, teeth grinding and horizontal nystagmus. These signs varied between individuals and did not occur in a fixed order. Shortly afterwards the animals became unresponsive and showed no voluntary motor activity, indicating adequate immobilization. Following naltrexone administration, the first signs of recovery occurred at a median of 2 min (range 1–3 min) and included coughing, vocalization, shivering and defecation.

The group median ratio, range (minimum–maximum), and interquartile range (IQR) of each variable post-administration of etorphine and naltrexone are presented in Supplementary Table S1 (cardiovascular variables), Supplementary Table S2 (EIT derived variables) and Supplementary Table S3 (EIT derived flow variables) of the Supplementary material.

Table 1 presents median and range (minimum-maximum) of PaO_2_ and PaCO_2_ values collected at baseline (BL) and 5 min after drug administration (T5) following etorphine and naltrexone injection.

**Table 1:** Arterial oxygen tension (PaO₂) and arterial carbon dioxide tension (PaCO₂) in mmHg measured 5 min before etorphine and 2 min before naltrexone administration (Pre) and 5 min after each respective drug (Post).

Etorphine	PaO_2_	PaCO_2_
Pre	98.1 (82.9–105.5)	41.7 (33.4–47.2)
Post	57.1 (26.9–93.6)	49.45 (40–61.3)

Naltrexone	PaO_2_	PaCO_2_
Pre	43.2 (30.5–61.9)	64.4 (57.6–68.9)
Post	63.5 (48.6–115.7)	46 (41.5–52.4)

Data are presented as median and range.

### 3.2 Effects of etorphine

#### 3.2.1 Cardiovascular parameters

For baseline and then each minute following etorphine and naltrexone injection, the median of the cardiovascular variables (HR, MAP, and MPAP) are presented in Table 2.

**Table 2:** Cardiovascular variables following etorphine and naltrexone injection.

Timepoint	HR	MAP	MPAP
Baseline (etorphine)	75	96	16
1	74	96	17
2	73	96	19
3	71	102	19
4	72	106	20
5	/	/	/
6	/	/	/
7	91	96	24
8	100	94	25
9	109	95	27
10	115	95	29
Baseline (naltrexone)	107	103	24
1	84	123	29
2	79	123	27
3	82	113	25
4	79	115	22
5	/	/	/
6	/	/	/
7	80	109	17
8	79	108	20
9	81	109	21
10	70	107	18

Data are expressed as the median of six sheep at each minute post-administration. Baseline is represented by the pooled median of the measurements obtained from the 5-min before drug injection. Measurements were unavailable 5 and 6 min after both drugs in five of the six sheep due to arterial blood gas sampling and catheter line flushing. Heart rate = HR (beats per minute - bpm); mean arterial pressure = MAP (mmHg); mean pulmonary arterial pressure = MPAP (mmHg).

Over the 10 min monitoring period, HR showed a mild non-significant overall increase (ratio 1.043). Individual timepoint analysis revealed substantial interindividual variability with no minute timeframe reaching statistical significance. Cardiac arrhythmias, however, were observed in 4 of 6 sheep following etorphine administration. Sinus tachyarrhythmia occurred in three animals, while one animal developed ventricular bigeminy. Arrhythmias developed at a median of 4 min (range 2–6 min) after etorphine injection.

Despite transient individual fluctuations, MAP remained stable (ratio of 1.056) with no significant deviations from baseline at any timepoint.

MPAP increased significantly with a median ratio of 1.420, corresponding to a 42% increase from baseline. Significant elevation was detected at minute 3 (*q* = 0.081) and persisted through minutes 7–10, indicating progressive pulmonary hypertension.

**Figure 3:**
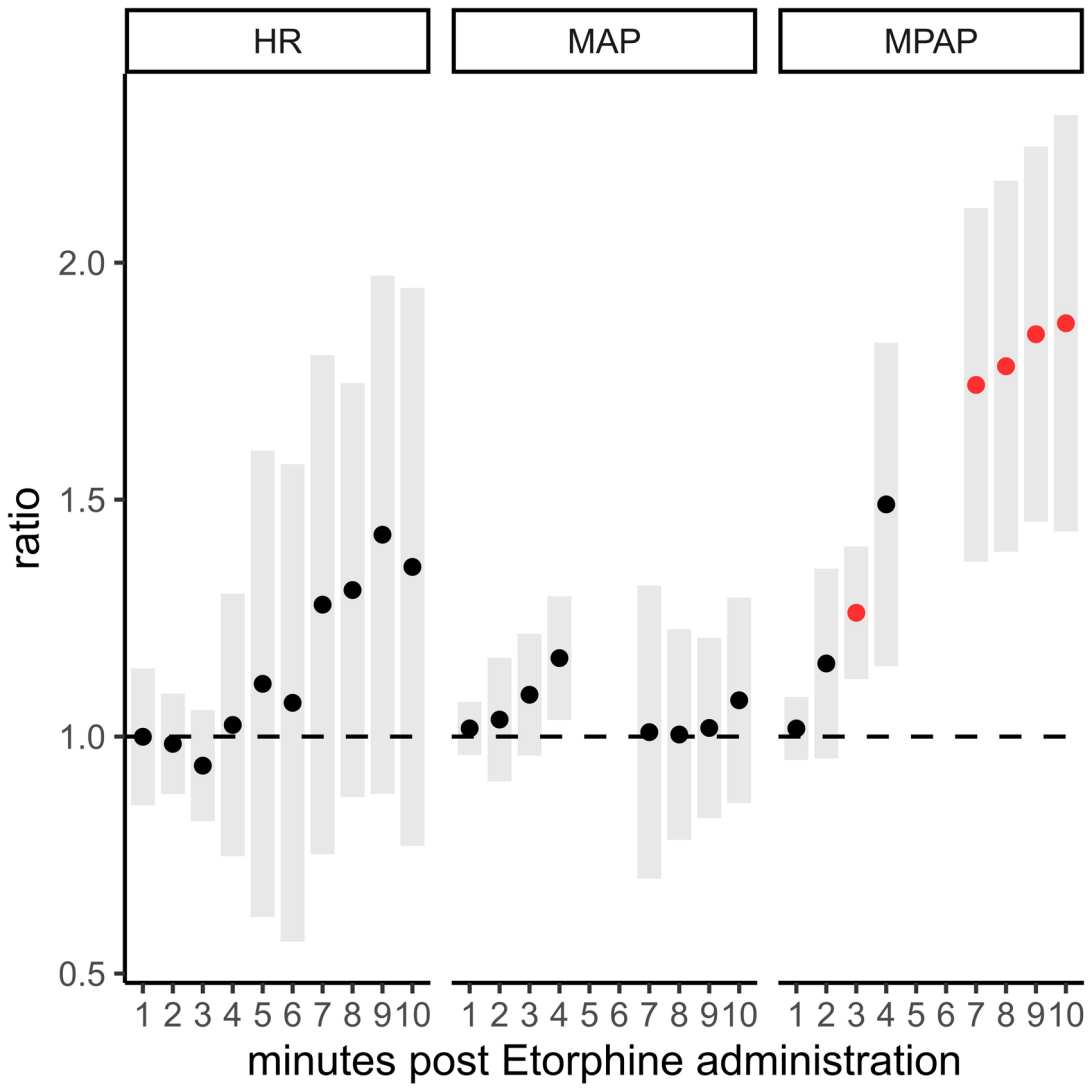
Estimated mean (dot) and 95% confidence intervals (gray bars) of cardiovascular variables expressed as ratios relative to pooled baseline (defined as the pooled mean of the measurements obtained from the 5-min before drug injection) following etorphine injection. The x-axis represents time after injection (minutes 1–10), and the y-axis represents the ratio to baseline. Black dots indicate no statistically significant difference from baseline, whereas red dots indicate significant differences at a global 10% False Discovery Rate (FDR). The dashed horizontal line represents the expectation under the null hypothesis of no difference. Measurements obtained at minute 5 and 6 for MAP and MPAP were due to arterial blood gas sampling and catheter line flushing. Heart rate = HR; mean arterial pressure = MAP; mean pulmonary arterial pressure = MPAP.

Cardiovascular values in Table 2 as well as their ratios in Figure 3 demonstrate the temporal dissociation between stable MAP and progressively increasing MPAP.

#### 3.2.2 EIT-derived variables

Over the 10 min observation period, TIV decreased significantly by 22.4% (ratio 0.776). Significant reductions were detected at minutes 5, 6 and 9 (*q* = 0.074) after etorphine injection, reflecting progressive hypoventilation.

Respiratory rate decreased significantly by 22.9% (ratio of 0.771). Significant decrease in RR was detected at minute 9 (*q* = 0.081).

Minute tidal impedance variation as a product of TIV and RR, decreased profoundly by 40.9% (ratio of 0.591). Significant reductions were detected at minutes 3 (*q* = 0.097), 6 (*q* = 0.089), and 10 (*q* = 0.074).

Inspiratory time showed a non-significant prolongation (ratio 1.185).

**Figure 4:**
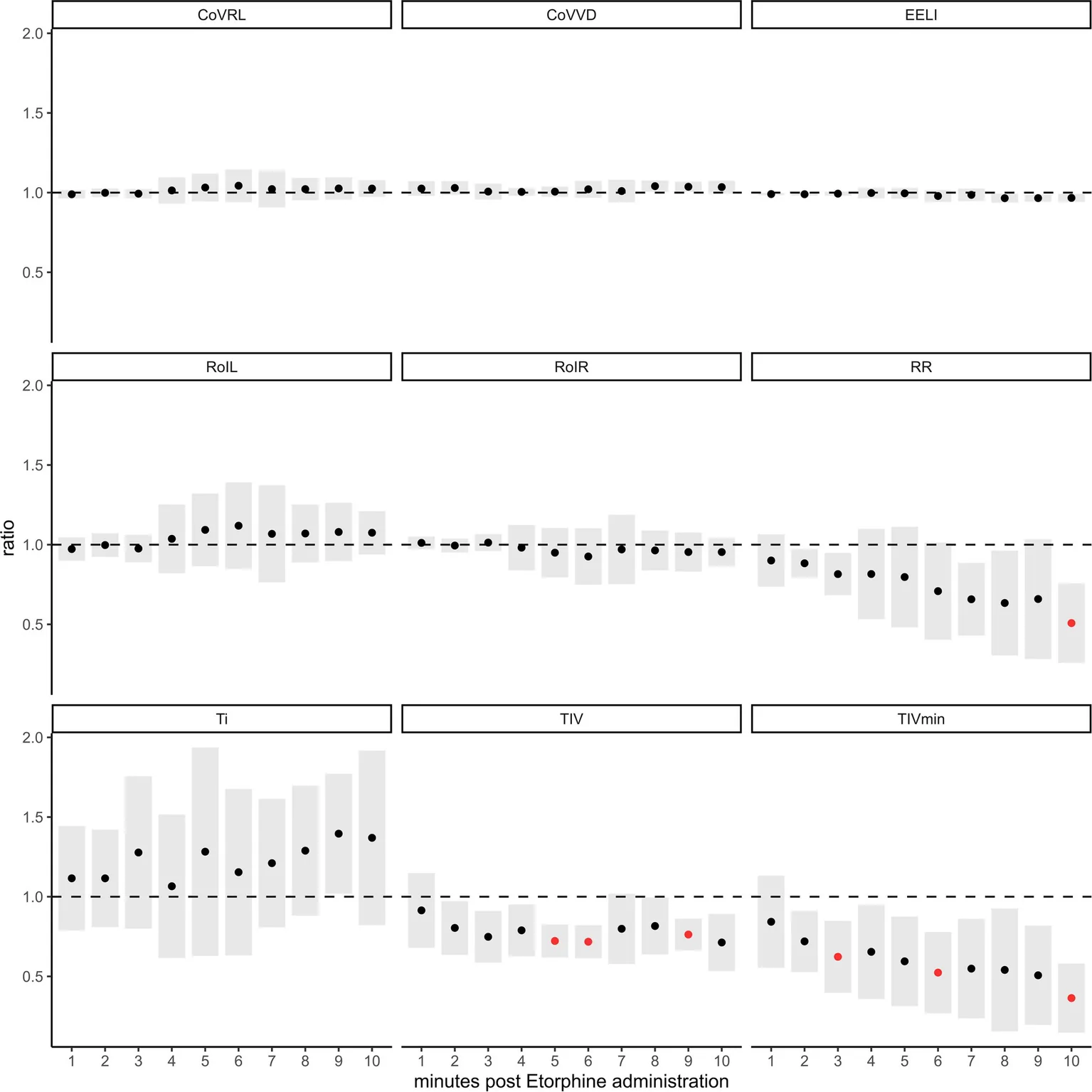
Estimated mean (dot) and 95% confidence intervals (gray bars) of EIT-derived variables expressed as ratios relative to pooled baseline (defined as the pooled mean of the measurements obtained from the 5-min before drug injection) following etorphine injection. The x-axis represents time after injection (minutes 1–10), and the y-axis represents the ratio to baseline. Black dots indicate no statistically significant difference from baseline, whereas red dots indicate significant differences at a global 10% False Discovery Rate (FDR). The dashed horizontal line represents the expectation under the null hypothesis of no difference. Center of ventilation right-to-left = CoVRL; center of ventilation ventral-to-dorsal = CoVVD; end-expiratory lung impedance = EELI; region of interest left lung = RoIL; region of interest right lung = RoIR; respiratory rate = RR; inspiratory time = Ti; tidal impedance variation = TIV; minute tidal impedance variation = TIV_MIN_.

Regional ventilation distribution variables (CoVRL, CoVVD, RoIR, RoIL) and EELI remained stable, with no significant changes detected at any timepoint (Figure 4).

#### 3.2.3 EIT-derived flow variables

**Figure 5:**
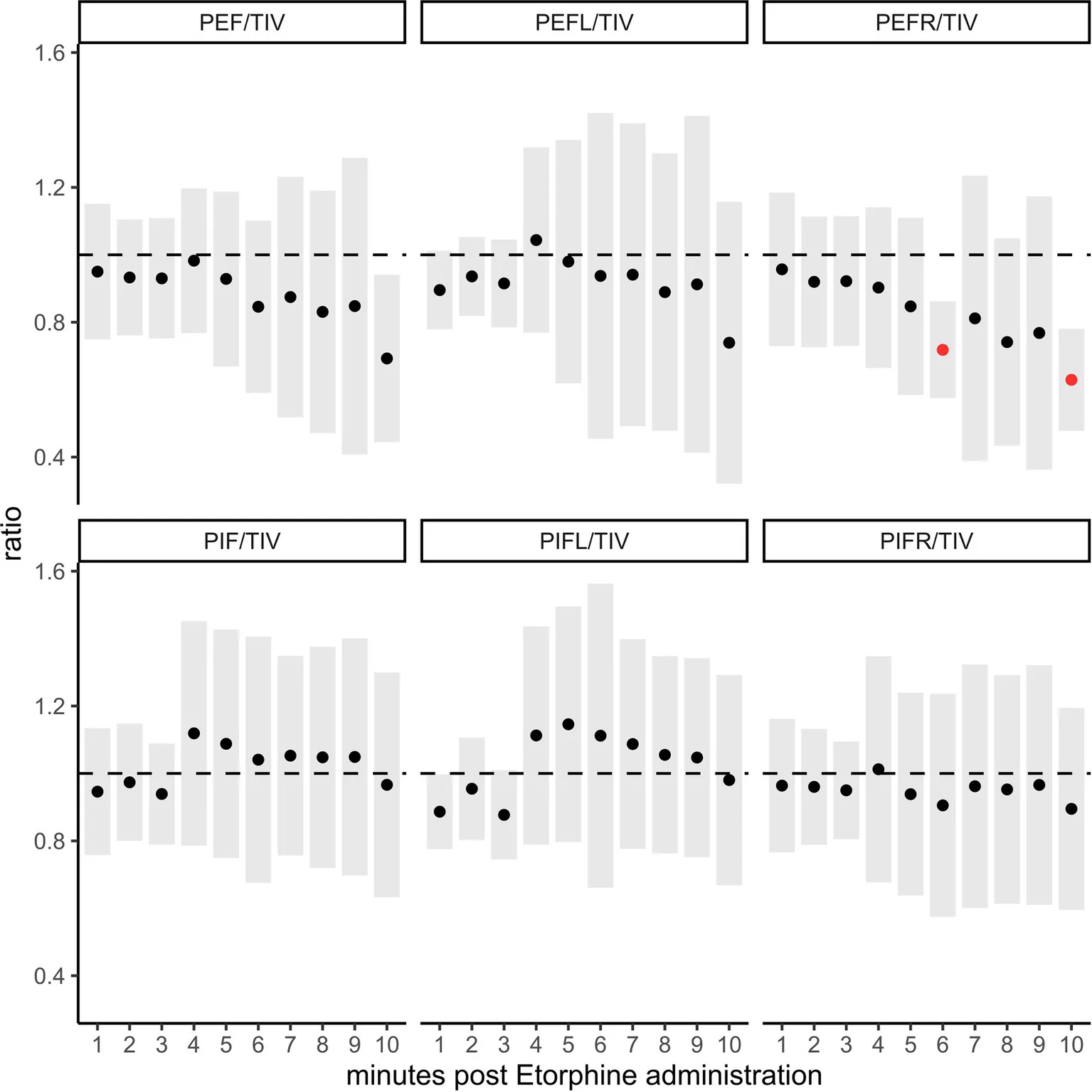
Estimated mean (dot) and 95% confidence intervals (gray bars) of tidal impedance variation normalized global and regional EIT-derived flow variables expressed as ratios relative to pooled baseline (defined as the pooled median of the measurements obtained from the 5-min before drug injection) following etorphine injection. The x-axis represents time after injection (minutes 1–10), and the y-axis represents the ratio to baseline. Black dots indicate no statistically significant difference from baseline, whereas red dots indicate significant differences at a global 10% False Discovery Rate (FDR). The dashed horizontal line represents the expectation under the null hypothesis of no difference. Global expiratory flow = PEF; left regional expiratory flow = PEFL; right regional expiratory flow = PEFR; global inspiratory flow = PIF; left regional inspiratory flow = PIFL; right regional inspiratory flow = PIFR. All EIT derived flow variables are expressed as normalized to tidal impedance variation (TIV).

Because TIV changed significantly, flow variables were normalized to TIV to account for alterations in tidal volume. Normalized inspiratory and expiratory flow variables are shown in Figure 5.

Peak expiratory flow of the right lung normalized to tidal impedance variation (PEFR/TIV) decreased significantly by 24.7% (ratio of 0.753). Significant reductions were detected at minutes 6 (*q* = 0.081) and 10 (*q* = 0.079).

Global peak inspiratory flow to TIV ratio (PIF/TIV) and all other regional flow parameters showed non-significant trends but did not reach statistical significance at any individual timepoint.

### 3.3 Effects of naltrexone

#### 3.3.1 Cardiovascular variables

Over the 10 min observation period after naltrexone, HR decreased significantly by 29.1% (ratio 0.709). Significant reduction was detected from minutes 1 to 9. No arrhythmias were detected in this phase.

After naltrexone, MAP showed a transient, significant increase during the first 2 min post injection returning to baseline levels by minute 3. The overall ratio of 0.940 did not indicate sustained deviation from baseline.

**Figure 6:**
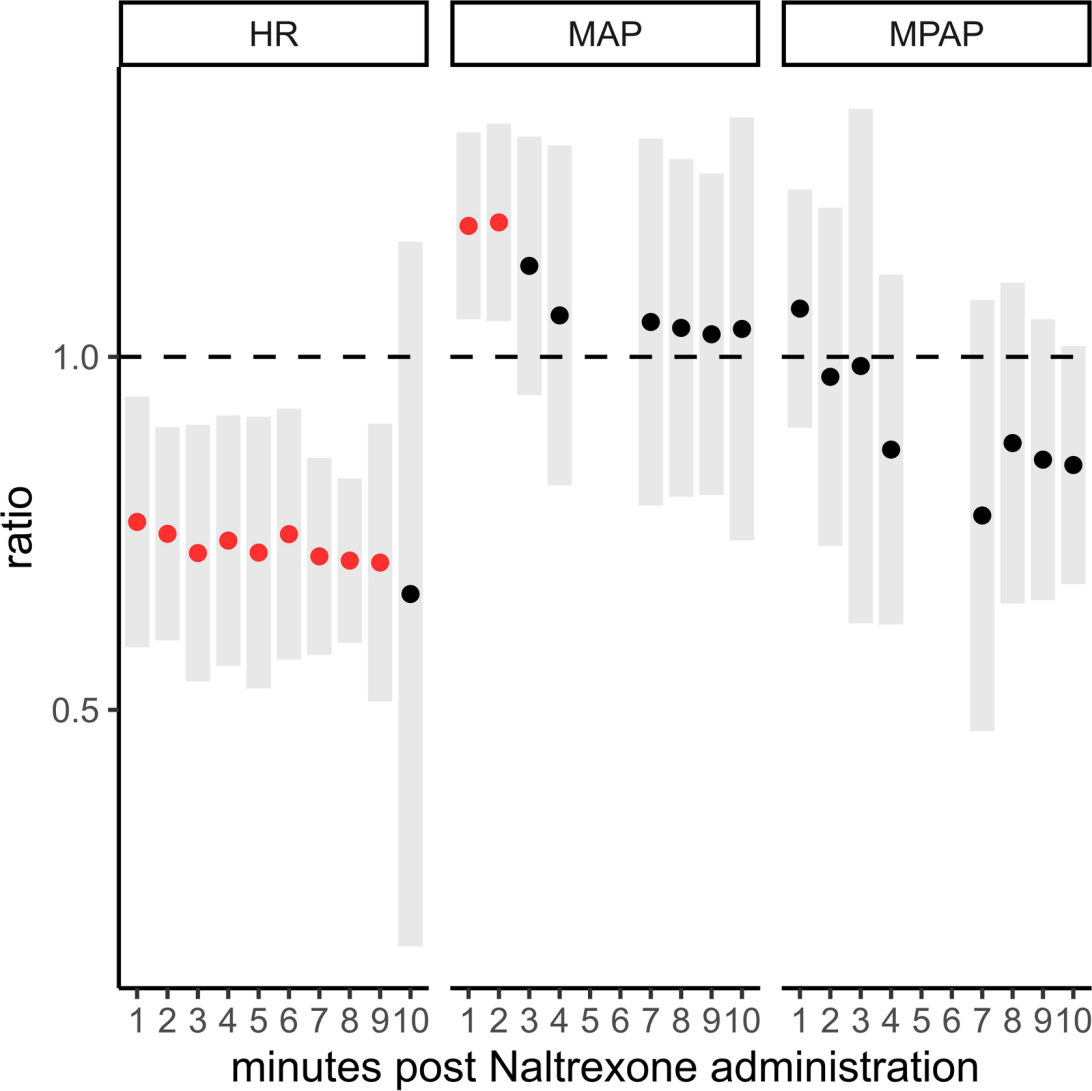
Estimated mean (dot) and 95% confidence intervals (gray bars) of cardiovascular variables expressed as ratios relative to pooled baseline (defined as the pooled mean of the measurements obtained from the 5-min before drug injection) following naltrexone injection. The x-axis represents time after injection (minutes 1–10), and the y-axis represents the ratio to baseline. Black dots indicate no statistically significant difference from baseline, whereas red dots indicate significant differences at a global 10% False Discovery Rate (FDR). The dashed horizontal line represents the expectation under the null hypothesis of no difference. Measurements obtained at minute 5 and 6 for MAP and MPAP were due to arterial blood gas sampling and catheter line flushing. Heart rate = HR; mean arterial pressure = MAP; mean pulmonary arterial pressure = MPAP.

A non-significant decrease was observed for MPAP (ratio 1.115), which did not reach statistical significance at any timepoint during the 10-min observation period (Figure 6).

#### 3.3.2 EIT-derived variables

Naltrexone produced rapid and marked improvement of ventilation.

A significant increase in TIV of 50.8% (ratio 1.508) was observed, with significant differences from baseline between minutes 4 to 9. This was accompanied by a marked increase in RR of 119.3% (ratio of 2.193) which was significant at all timepoints from minute 1 to 10. As a result, TIV_MIN_ increased profoundly by 223.6% (ratio 3.236). Significant increase was detected throughout the entire measurement period. In parallel, Ti decreased (ratio 0.681), with significant changes at all timepoints except minute 3.

**Figure 7:**
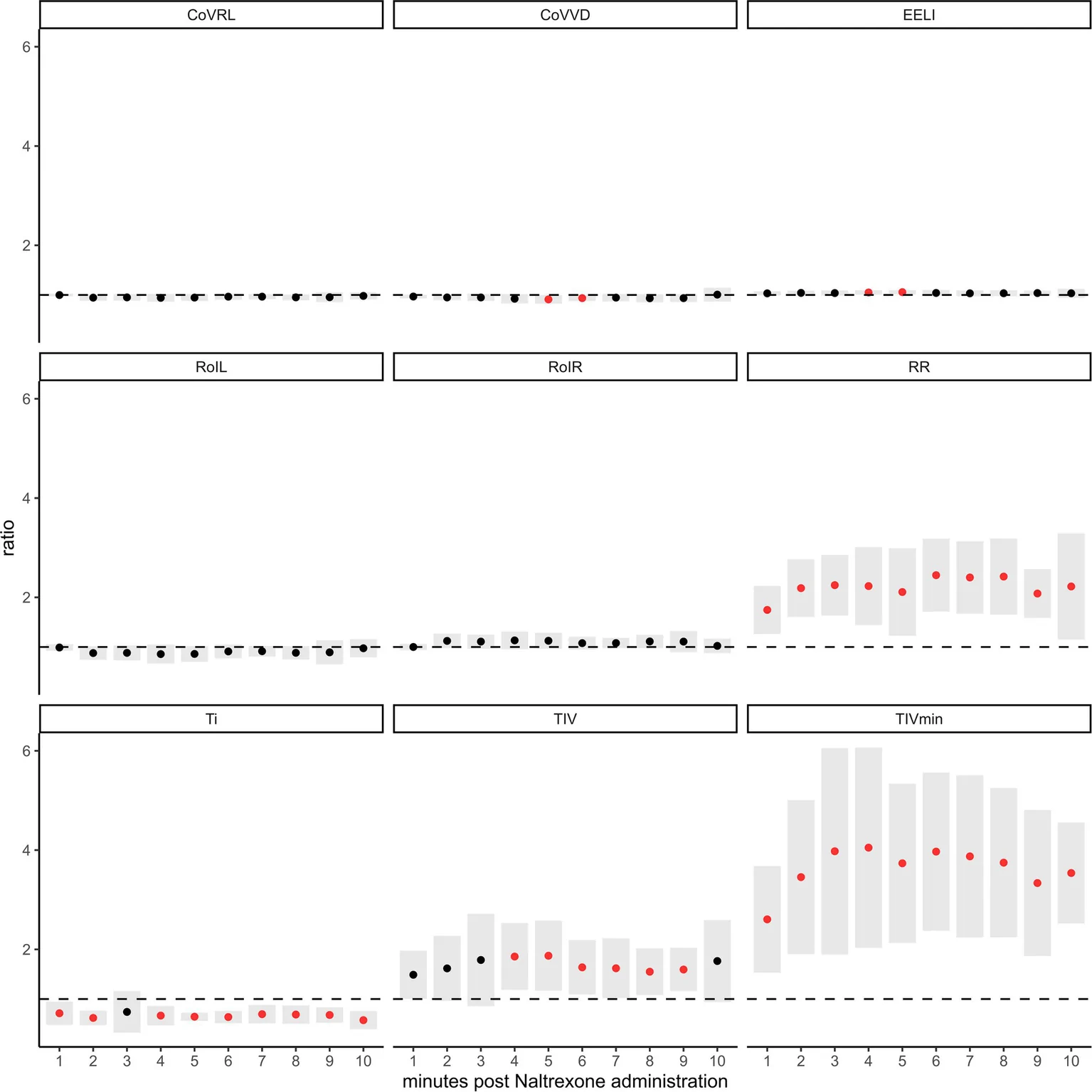
Estimated mean (dot) and 95% confidence intervals (gray bars) of EIT-derived variables expressed as ratios relative to pooled baseline (defined as the pooled mean of the measurements obtained from the 5-min before drug injection) following naltrexone injection. The x-axis represents time after injection (minutes 1–10), and the y-axis represents the ratio to baseline. Black dots indicate no statistically significant difference from baseline, whereas red dots indicate significant differences at a global 10% False Discovery Rate (FDR). The dashed horizontal line represents the expectation under the null hypothesis of no difference. Center of ventilation right-to-left = CoVRL; center of ventilation ventral-to-dorsal = CoVVD; end-expiratory lung impedance = EELI; region of interest left lung = RoIL; region of interest right lung = RoIR; respiratory rate = RR; inspiratory time = Ti; tidal impedance variation = TIV; minute tidal impedance variation = TIV_MIN._

Regional ventilation distribution parameters (CoVRL, CoVVD, RoIR, RoIL) and EELI remained stable during naltrexone reversal. Nevertheless, CoVVD showed a decrease (ratio 0.940), with transient significant differences at minutes 5 and 6. Similarly, EELI showed an increase (ratio 1.041), with significance detected at minutes 4 and 5. Overall, these changes were small and not sustained (Figure 7).

#### 3.3.3 EIT-derived flow variables

**Figure 8:**
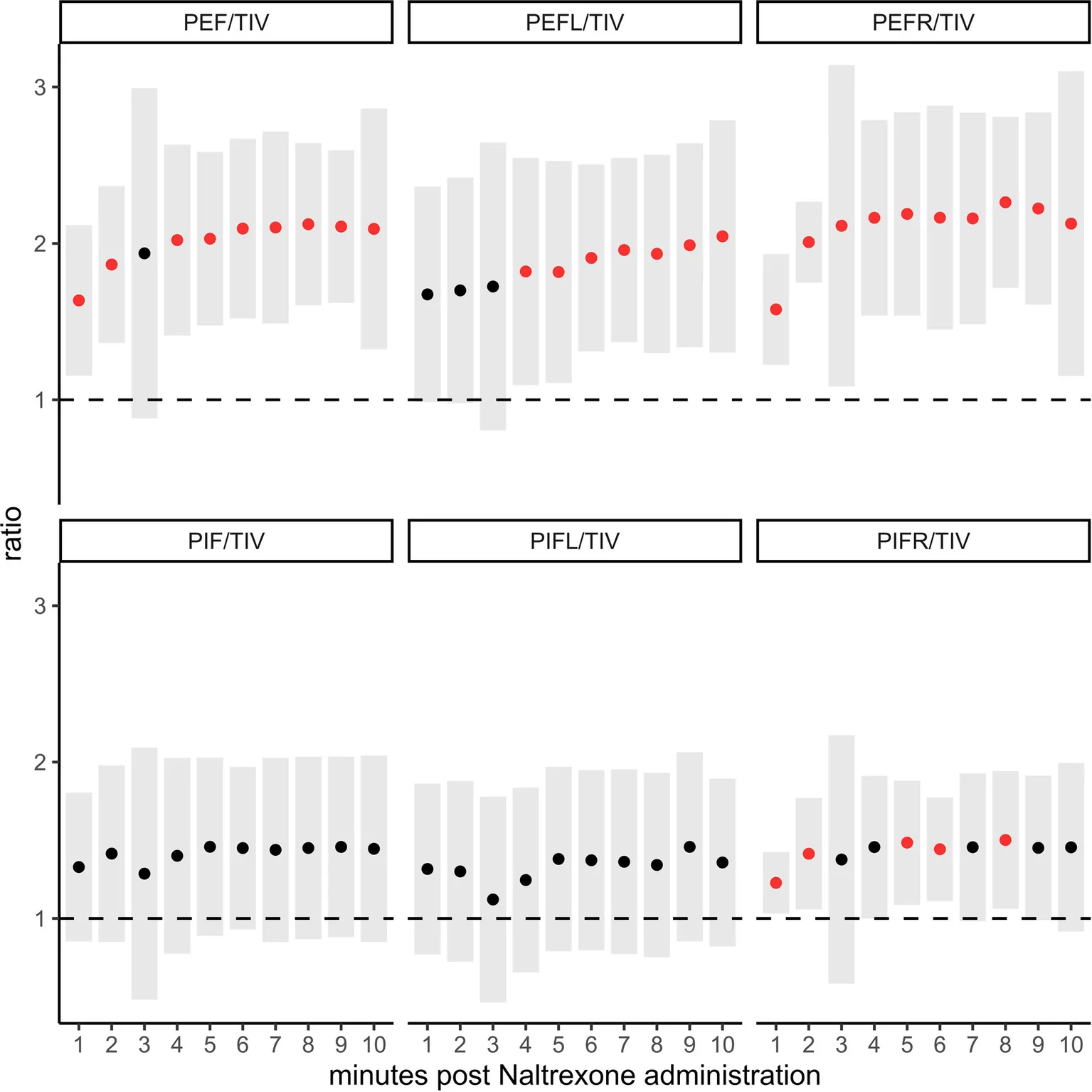
Estimated mean (dot) and 95% confidence intervals (gray bars) of tidal impedance variation normalized global and regional EIT-derived flow variables expressed as ratios relative to pooled baseline (defined as the pooled median of the measurements obtained from the 5-min before drug injection) following naltrexone injection. The x-axis represents time after injection (minutes 1–10), and the y-axis represents the ratio to baseline. Black dots indicate no statistically significant difference from baseline, whereas red dots indicate significant differences at a global 10% False Discovery Rate (FDR). The dashed horizontal line represents the expectation under the null hypothesis of no difference. Global expiratory flow = PEF; left regional expiratory flow = PEFL; right regional expiratory flow = PEFR; global inspiratory flow = PIF; left regional inspiratory flow = PIFL; right regional inspiratory flow = PIFR. All EIT derived flow variables are expressed as normalized to tidal impedance variation (TIV).

Normalized inspiratory and expiratory flow variables are shown in Figure 8.

The largest increase is in PEF/TIV, rising by 119% from baseline (ratio 2.186). Significant elevations were detected at minutes 1–10, except for minute 3. In the right lung, PEFR/TIV increased by 102% from baseline (ratio 2.017), with significant increase observed at minutes 1–10 while in the left lung, PEFL/TIV increased by 91% (ratio 1.914), with significant differences detected at minutes 4–10.

Inspiratory flow indices showed smaller changes than expiratory variables with PIF/TIV and PIFL/TIV increasing by 28% (ratio 1.275) and 18% (ratio 1.182), respectively and no significant deviations from baseline at any timepoint. In contrast, PIFR/TIV increased by 32% (ratio 1.316), with significant elevations at minutes 1, 2, 5, 6, and 8 following naltrexone administration.

## 4 Discussion

This study describes the minute-by-minute cardiorespiratory changes during the transition from consciousness to etorphine immobilization and reversal with naltrexone in sheep as a model for wild ungulates. The rapid onset of etorphine was consistent with previous studies, with clinical signs developing within 1–3 min (2, 7). Using continuous invasive haemodynamic monitoring together with EIT, a clear temporal pattern emerged: in contrast to our first hypothesis, mean arterial pressure did not change. On the other hand, pulmonary hypertension developed first (minute 3), followed by progressive hypoventilation and hypoxemia (minutes 5–9), consistent with our first hypothesis. However, indices describing regional ventilation distribution and end-expiratory lung impedance remained largely unchanged. Although naltrexone restored ventilation from the first minute after injection, pulmonary arterial pressure and hypoxemia did not normalize within the observation period, suggesting that pulmonary vascular dysfunction may contribute to the severe hypoxemia associated with etorphine immobilization.

### 4.1 Cardiovascular effects

The earliest physiological alteration detected following etorphine administration was a significant increase in mean pulmonary arterial pressure, which is a well-recognized consequence of immobilization with ultra potent opioids. In goats, etorphine administration produces rapid pulmonary hypertension accompanied by severe hypoxemia (4, 28, 29). Similar pulmonary vascular responses have also been reported in large wildlife species such as white rhinoceroses (*Ceratotherium simum*) (30).

The mechanisms underlying opioid-induced pulmonary hypertension are likely multifactorial. Ultra-potent opioids may induce sympathetic activation and catecholamine release, increasing pulmonary vascular tone (1, 6). In addition, hypoxemia developing during immobilization may further aggravate pulmonary vasoconstriction through hypoxic pulmonary vascular responses (4). Concurrent hypercapnia and respiratory acidosis, both commonly observed during etorphine induced ventilatory depression, may further increase pulmonary vascular resistance through hypercapnic pulmonary vasoconstriction and may potentiate hypoxemia induced pulmonary vasoconstriction (4, 31). Previous studies have suggested that pulmonary hypertension contributes substantially to impaired oxygenation during etorphine immobilization. Proposed mechanisms include ventilation–perfusion mismatch, reduced pulmonary capillary transit time, and impaired alveolar–capillary gas exchange associated with pulmonary vascular hypertension (4, 6, 30). In addition, severe elevations in pulmonary vascular pressure may contribute to pulmonary vascular dysfunction. Previous pulmonary physiology literature has shown that marked increases in pulmonary vascular pressure can induce hydrostatic stress injury of the alveolar-capillary membrane and increase vascular permeability, potentially impairing gas exchange (32).

Although systemic hypertension and tachycardia are often reported with etorphine (1–3, 6), this pattern was not consistently observed here, instead mean arterial pressure and heart rate remained relatively stable despite the marked increase in mean pulmonary arterial pressure. One possible explanation is the early development of cardiac arrhythmias in our sheep (median 4 min, range 2–6 min), overlapping with the onset of pulmonary hypertension (minute 3). We speculate that these arrhythmias were precipitated by the rapid rise in pulmonary arterial pressure and, in combination with the elevated pulmonary vascular load, may have reduced cardiac output. This reduction in cardiac output could explain why systemic arterial pressure did not increase in our study, in contrast to findings reported in rhinoceroses (6).

### 4.2 Respiratory effects

EIT is increasingly used in veterinary medicine to evaluate ventilation distribution and respiratory mechanics across species (12–18), although most studies have been conducted under stable or controlled anesthetic conditions. In field settings, the early phase of immobilization is rarely monitored because animals cannot be approached safely during induction. Continuous respiratory monitoring using EIT in an instrumented model therefore provides a unique opportunity to characterise physiological events during this otherwise inaccessible phase of immobilisation.

Following the early pulmonary vascular response, etorphine induced progressive ventilatory depression, reflected by reductions in tidal impedance variation and respiratory rate. Because tidal impedance variation correlates with tidal volume (20, 21), this indicates a substantial reduction in tidal ventilation.

In the present study, reductions in tidal ventilation became significant 5 min before respiratory rate decreased, suggesting that suppression of inspiratory effort and respiratory drive may precede bradypnea during etorphine immobilization. Classically, opioid induced respiratory depression is primarily characterized by impaired respiratory rhythm generation and reduced respiratory frequency mediated through *μ*-opioid receptor effects within the pre-Bötzinger complex (33–35). However, the present study evaluated the acute transition from consciousness to immobilization after administration of an ultra-potent opioid in awake animals. During this early transition, respiratory rate may initially have been partially maintained by direct etorphine induced arousal and sympathetic activation, whereas compensatory chemoreceptor stimulation would be expected to increase as hypercapnia and hypoxemia developed. However, this compensatory drive may have been progressively outweighed by opioid induced central respiratory depression. Consequently, reductions in inspiratory effort and breath depth may have become detectable before reductions in respiratory frequency. The continuous minute by minute EIT analysis performed in the present study may therefore have allowed characterization of these early transitional ventilatory changes during immobilization. The marked reduction in minute tidal impedance variation observed here indicates a substantial decrease in effective minute ventilation during the early immobilization phase and supports previous observations that ventilatory depression represents a major contributor to impaired gas exchange during etorphine immobilization (28, 36, 37). Similar ventilatory depression has been reported in several species immobilized with etorphine, including goats and antelope, where reduced ventilation contributes significantly to hypoxemia and hypercapnia (7, 28, 36).

Arterial blood gas analysis confirmed hypoventilation by hypercapnia. However, the severe hypoxaemia observed following etorphine administration cannot be attributed solely to hypoventilation. Within the classical framework of the five principal mechanisms of hypoxaemia —reduced inspired oxygen fraction, hypoventilation, diffusion limitation, ventilation–perfusion mismatch, and right-to-left shunt (38)— it is likely that additional processes, beyond hypoventilation, contributed to the observed impairment in oxygenation. Reduced inspired oxygen fraction can be excluded, as animals were breathing room air under controlled experimental conditions. Diffusion limitation appears unlikely, as it would be expected to cause a shift in ventilation distribution due to increased lung water (14, 17). Likewise, a substantial right-to-left shunt from alveolar collapse is improbable, as this would be reflected by altered ventilation distribution and reduced end-expiratory lung impedance. However, EIT-derived indices, including centre of ventilation and end-expiratory lung impedance, remained largely unchanged, indicating that etorphine-induced hypoxaemia occurred without significant redistribution of ventilation or lung collapse within the measured thoracic plane. Similar findings have been reported in etorphine immobilized white rhinoceroses, where severe hypoxemia occurs despite preserved lung aeration (39). The standardized supported sternal positioning used in this study might have contributed to the stability of ventilation distribution observed with EIT.

Therefore, the combination of stable ventilation distribution, significant pulmonary hypertension, and marked hypoxemia suggests that ventilation–perfusion mismatch represents a possible contributing mechanism of impaired gas exchange in this model. Increased pulmonary pressures alter perfusion distribution, reducing blood flow to well-ventilated lung regions and impairing oxygen uptake. This interpretation is consistent with previous studies, which have demonstrated that pulmonary hypertension contributes substantially to hypoxemia in etorphine-immobilized animals (4, 30). Under these conditions, hypoxemia appears to be driven predominantly by pulmonary perfusion derangements rather than by primary alterations in ventilation or alveolar collapse.

Additionally, analysis of EIT-derived flow variables showed only minor changes when normalized to TIV, suggesting that the primary respiratory effect of etorphine in this model was a reduction in ventilatory drive rather than a substantial alteration in airway resistance or expiratory flow limitation.

### 4.3 Effects of naltrexone

Administration of naltrexone resulted in rapid reversal of ventilatory depression indicated by an increase in respiratory rate, tidal impedance and minute tidal impedance variation. This rapid recovery is consistent with the pharmacological action of naltrexone as a competitive opioid receptor antagonist that displaces etorphine from *μ*-opioid receptors and restores respiratory rhythm generation (34). The immediate increase in respiratory rate likely reflects both pharmacological reversal of central respiratory depression and a combined stimulatory effect of residual hypercapnia, hypoxemia, and arousal related stress. Rapid recovery of alveolar ventilation was confirmed by a marked reduction in PaCO_2_ 5 minutes after reversal.

Despite the immediate improvement in ventilation, pulmonary pressures showed a slower and incomplete recovery. Although mean pulmonary arterial pressure decreased after reversal, normalization or a statistically significant decrease was not observed within the 10-min observation period. This may reflect the fact that opioid induced pulmonary hypertension is not solely driven by hypoventilation but also involves pulmonary vascular mechanisms which may persist beyond restoration of ventilatory drive after naltrexone (4, 29, 30). On the other side, mean arterial pressure increased transiently during the first 2 min before returning to baseline. No arrhythmias were observed following reversal, but heart rate decreased, likely reflecting the attenuation of cardiac opioid induced sympathetic stimulation (6, 7).

Oxygenation improved but remained below pre-etorphine baseline values. This dissociation between rapid recovery of ventilation (within 1 min) and delayed improvement in oxygenation, together with persistently elevated mean pulmonary arterial pressure, suggests that pulmonary vascular effects persist beyond reversal of central respiratory depression. Previous studies have similarly proposed that etorphine-induced hypoxemia is not solely attributable to hypoventilation but is markedly influenced by pulmonary hypertension and impaired gas exchange at the alveolar level. In goats, hypoxemia was shown to be more severe than expected from hypoventilation alone and was strongly associated with increases in pulmonary arterial pressure, indicating a primary role of pulmonary vascular alterations in limiting oxygen transfer (4). However, these observations were derived from discrete timepoint measurements and thus provide a broader overview of the response. In that study, pulmonary hypertension appeared to resolve within the observation period. In contrast, the present data, analyzed minute-by-minute, allow a more detailed assessment of the temporal changes and demonstrate that pulmonary vascular alterations persist despite rapid restoration of ventilatory variables, supporting a predominant role of perfusion-related mechanisms.

EIT-derived indices of ventilation distribution remained stable during reversal, with only minor and transient changes in center of ventilation ventral-to-dorsal (minutes 5–6) and end-expiratory lung impedance (minutes 4–5), suggesting no meaningful redistribution of ventilation. To the authors knowledge, this has not been described so far with naltrexone.

Normalized global and regional expiratory flow indices increased markedly from minute 1 onwards, whereas global and regional inspiratory flow indices showed only minor changes. This pattern is likely explained by the pronounced increase in RR rather than changes in airway resistance and narrowing of airways.

### 4.4 Limitations

Several limitations should be considered when interpreting these findings. The relatively small number of animals limits statistical power and the ability to detect subtle physiological effects.

The sheep in this study were part of larger trial and included data stem solely from the control group. Multiple exposure to drugs might have influenced physiological responses although a washout period of at least 4 weeks was implemented and sheep were deemed healthy prior to each anesthetic event.

A Swan–Ganz catheter was used to allow continuous monitoring of pulmonary arterial pressure. Although this technique enables cardiac output measurement via thermodilution, this was not performed as repeated injections would have interrupted continuous haemodynamic recording within the 10 min observation period, which was a primary objective of the study. Similarly, arterial blood gas sampling led to missing data at minutes 5 and 6 after both etorphine and naltrexone.

Sheep were used as a model species to allow detailed physiological measurements that are not feasible in free-ranging wildlife. Although sheep share physiological similarities with other large herbivorous ungulates and have been used as experimental models in immobilization studies, species-specific differences in opioid sensitivity may exist.

In addition, EIT measurements were obtained from a single thoracic plane and evaluated ventilation only. Simultaneous perfusion EIT was not performed. Consequently, diffuse pulmonary alterations, ventilation–perfusion mismatch, microvascular injury, subtle pulmonary edema, diffusion impairment, or intrapulmonary shunting occurring without substantial regional ventilation redistribution or outside the measured thoracic plane may not have been detected.

Finally, substantial interindividual variability (reflected by wide confidence intervals) was observed in several physiological variables during the etorphine phase compared with the relatively consistent responses following naltrexone administration. Such variability likely reflects the complex interaction between opioid pharmacodynamics and the physiological stress response, including sympathetic activation and catecholamine release, as well as species-specific and individual sensitivity to potent opioids, and may have influenced the observed effects.

## 5 Conclusion

Etorphine immobilization induced a rapid and distinct cardiorespiratory sequence, with early pulmonary hypertension followed by progressive ventilatory depression. Despite marked reductions in ventilation, EIT demonstrated stable ventilation distribution within the measured thoracic plane, suggesting that altered pulmonary perfusion and ventilation–perfusion mismatch may contribute importantly to etorphine induced hypoxemia.

Following naltrexone, ventilation recovered rapidly, whereas pulmonary arterial pressure and oxygenation failed to recover within the same timeframe, further supporting a primary role of altered pulmonary perfusion in the development of hypoxemia during etorphine immobilization.

Improved understanding of the temporal development of pulmonary vascular and ventilatory alterations during etorphine immobilization may contribute to safer monitoring and management of opioid-based immobilization protocols in wildlife and large animal species.

## Data Availability

The raw data supporting the conclusions of this article will be made available by the authors, without undue reservation.
