# Correction: Early cardiovascular and respiratory changes after etorphine immobilization and naltrexone reversal in sheep

**DOI:** 10.3389/fvets.2026.1981732

**Published:** 2026-09-16

**Authors:** Frontiers Production Office

**Affiliations:** Frontiers Media SA, Lausanne, Switzerland

**Keywords:** electrical impedance tomography, opioid induced pulmonary hypertension, opioids antagonist, ultra potent opioids, ungulates

The funder University of Veterinary Medicine Vienna was erroneously omitted from the **Funding** statement. The correct Funding statement reads:

“The author(s) declared that financial support was received for this work and/or its publication. This work was funded by a scholarship of the Chulabhorn Royal Academy, Bangkok, Thailand. Open access funding provided by University of Veterinary Medicine Vienna.”

The original version of this article has been updated.

