# Supplementary material for: Early cardiovascular and respiratory changes after etorphine immobilization and naltrexone reversal in sheep
Source: Front Vet Sci. 2026 Jul 13;13:1850859. doi: 10.3389/fvets.2026.1850859 (PMC13402195; doi:10.3389/fvets.2026.1850859)
Supplement: Supplementary file 1 [file Data_Sheet_1.PDF]

Supplementary Material

Supplementary table S1: Cardiovascular variables following etorphine and naltrexone administration, expressed as post-treatment-to-baseline ratios.
Data are presented as ratios calculated as the median value during the 10-minute period after drug injection divided by the corresponding baseline median value (baseline is represented by the pooled median of the measurements obtained from the 5-minutes before drug injection). A ratio of 1 indicates no change from baseline; values >1 indicate an increase, and values <1 indicate a decrease relative to baseline. For each variable, the table reports the group median ratio, range (minimum–maximum), and interquartile range (IQR) of the ratio. Heart rate = HR; mean pulmonary arterial pressure = MPAP; mean arterial pressure = MAP.

**Etorphine**	**Ratio**	**Range**	**IQR**	**Naltrexone**	**Ratio**	**Range**	**IQR**
**HR**	1.043	(0.64–2.06)	(0.89–1.37)	**HR**	0.709	(0.27–1.06)	(0.63–0.81)
**MPAP**	1.420	(0.97–2.38)	(1.14–1.82)	**MPAP**	0.940	(0.52–1.31)	(0.70–1.05)
**MAP**	1.056	(0.62–1.47)	(0.97–1.15)	**MAP**	1.115	(0.75–1.39)	(0.92–1.20)

Supplementary table S2: Electrical impedance tomography (EIT) variables following etorphine and naltrexone administration, expressed as post-treatment-to-baseline ratios.
Data are presented as ratios calculated as the median value during the 10-minute period after drug injection divided by the corresponding baseline median value (baseline is represented by the pooled median of the measurements obtained from the 5-minutes before drug injection). A ratio of 1 indicates no change from baseline; values >1 indicate an increase, and values <1 indicate a decrease relative to baseline. For each variable, the table reports the group median ratio, range (minimum–maximum), and interquartile range (IQR) of the ratio. Center of ventilation right-to-left = CoVRL; center of ventilation ventral-to-dorsal = CoVVD; region of interest right lung = RoIR; region of interest left lung = RoIL; inspiratory time = Ti; tidal impedance variation = TIV; respiratory rate =RR; minute tidal impedance variation = TIV_{MIN}, and end-expiratory lung impedance = EELI.

**Etorphine**	**Ratio**	**Range**	**IQR**	**Naltrexone**	**Ratio**	**Range**	**IQR**
**CoVRL**	1.005	(0.87–1.21)	(0.99–1.02)	**CoVRL**	0.985	(0.83–1.08)	(0.91–1.00)
**CoVVD**	1.016	(0.89–1.10)	(1.00–1.05)	**CoVVD**	0.940	(0.79–1.17)	(0.91–0.99)
**RoIR**	0.990	(0.63–1.28)	(0.96–1.02)	**RoIR**	1.036	(0.86–1.40)	(0.99–1.20)
**RoIL**	1.015	(0.68–1.58)	(0.97–1.07)	**RoIL**	0.938	(0.59–1.23)	(0.79–1.01)
**Ti**	1.185	(0.58–2.40)	(0.96–1.55)	**Ti**	0.681	(0.33–1.31)	(0.59–0.75)
**TIV**	0.776	(0.49–1.19)	(0.68–0.86)	**TIV**	1.508	(0.88–2.99)	(1.23–2.12)
**RR**	0.771	(0.22–1.29)	(0.55–0.92)	**RR**	2.193	(1.00–3.57)	(1.78–2.72)
**TIV_{MIN}**	0.591	(0.17–1.22)	(0.39–0.80)	**TIV_{MIN}**	3.236	(1.33–6.83)	(2.49–4.70)
**EELI**	0.983	(0.94–1.05)	(0.97–1.00)	**EELI**	1.041	(0.94–1.14)	(1.00–1.07)

Supplementary table S3: Electrical impedance tomography (EIT) derived flow variables following etorphine and naltrexone administration, expressed as post-treatment-to-baseline ratios.
Data are presented as ratios calculated as the median value during the 10-minute period after drug injection divided by the corresponding baseline median value (baseline is represented by the pooled median of the measurements obtained from the 5-minutes before drug injection). A ratio of 1 indicates no change from baseline; values >1 indicate an increase, and values <1 indicate a decrease relative to baseline. For each variable, the table reports the group median ratio, range (minimum–maximum), and interquartile range (IQR) of the ratio.
Global inspiratory flow = PIF; global expiratory flow =PEF; right regional inspiratory flow = PIFR; right regional expiratory flow = PEFR; left regional inspiratory flow = PIFL; left regional expiratory flow = PEFL.
All EIT derived flow variables are expressed as normalized to tidal impedance variation (TIV).

**Etorphine**	**Ratio**	**Range**	**IQR**	**Naltrexone**	**Ratio**	**Range**	**IQR**
**PIF/TIV**	0.956	(0.72–1.71)	(0.85–1.14)	**PIF/TIV**	1.275	(0.72–2.46)	(1.03–1.81)
**PEF/TIV**	0.878	(0.41–1.58)	(0.70–1.00)	**PEF/TIV**	2.186	(0.97–3.07)	(1.77–2.37)
**PIFR/TIV**	0.884	(0.63–1.55)	(0.76–1.09)	**PIFR/TIV**	1.316	(0.75–2.41)	(1.15–1.75)
**PEFR/TIV**	0.753	(0.45–1.61)	(0.65–0.94)	**PEFR/TIV**	2.017	(1.05–3.43)	(1.68–2.67)
**PIFL/TIV**	0.958	(0.69–1.60)	(0.84–1.18)	**PIFL/TIV**	1.182	(0.68–2.37)	(0.98–1.57)
**PEFL/TIV**	0.874	(0.45–1.70)	(0.71–1.03)	**PEFL/TIV**	1.914	(0.63–2.84)	(1.46–2.37)
