# Supplementary material for: Early cardiovascular and respiratory changes after etorphine immobilization and naltrexone reversal in sheep
Source: Front Vet Sci. 2026 Jul 13;13:1850859. doi: 10.3389/fvets.2026.1850859 (PMC13402195; doi:10.3389/fvets.2026.1850859)
Supplement: Supplementary file 2 [file Table_1.DOCX]

Supplementary Material

Supplementary table S1: Cardiovascular variables following etorphine and naltrexone administration, expressed as post-treatment-to-baseline ratios.
Data are presented as ratios calculated as the median value during the 10-minute period after drug injection divided by the corresponding baseline median value (baseline is represented by the pooled median of the measurements obtained from the 5-minutes before drug injection). A ratio of 1 indicates no change from baseline; values >1 indicate an increase, and values <1 indicate a decrease relative to baseline. For each variable, the table reports the group median ratio, range (minimum–maximum), and interquartile range (IQR) of the ratio.
Heart rate = HR; mean pulmonary arterial pressure = MPAP; mean arterial pressure = MAP.

| **Etorphine** | **Ratio** | **Range** | **IQR** | **Naltrexone** | **Ratio** | **Range** | **IQR** |
| --- | --- | --- | --- | --- | --- | --- | --- |
| **HR** | 1.043 | (0.64–2.06) | (0.89–1.37) | **HR** | 0.709 | (0.27–1.06) | (0.63–0.81) |
| **MPAP** | 1.420 | (0.97–2.38) | (1.14–1.82) | **MPAP** | 0.940 | (0.52–1.31) | (0.70–1.05) |
| **MAP** | 1.056 | (0.62–1.47) | (0.97–1.15) | **MAP** | 1.115 | (0.75–1.39) | (0.92–1.20) |

Supplementary table S2: Electrical impedance tomography (EIT) variables following etorphine and naltrexone administration, expressed as post-treatment-to-baseline ratios.
Data are presented as ratios calculated as the median value during the 10-minute period after drug injection divided by the corresponding baseline median value (baseline is represented by the pooled median of the measurements obtained from the 5-minutes before drug injection). A ratio of 1 indicates no change from baseline; values >1 indicate an increase, and values <1 indicate a decrease relative to baseline. For each variable, the table reports the group median ratio, range (minimum–maximum), and interquartile range (IQR) of the ratio.
Center of ventilation right-to-left = CoVRL; center of ventilation ventral-to-dorsal = CoVVD; region of interest right lung = RoIR; region of interest left lung = RoIL; inspiratory time = Ti; tidal impedance variation = TIV; respiratory rate =RR; minute tidal impedance variation = TIV_MIN_, and end-expiratory lung impedance = EELI.

| **Etorphine** | **Ratio** | **Range** | **IQR** | **Naltrexone** | **Ratio** | **Range** | **IQR** |
| --- | --- | --- | --- | --- | --- | --- | --- |
| **CoVRL** | 1.005 | (0.87–1.21) | (0.99–1.02) | **CoVRL** | 0.985 | (0.83–1.08) | (0.91–1.00) |
| **CoVVD** | 1.016 | (0.89–1.10) | (1.00–1.05) | **CoVVD** | 0.940 | (0.79–1.17) | (0.91–0.99) |
| **RoIR** | 0.990 | (0.63–1.28) | (0.96–1.02) | **RoIR** | 1.036 | (0.86–1.40) | (0.99–1.20) |
| **RoIL** | 1.015 | (0.68–1.58) | (0.97–1.07) | **RoIL** | 0.938 | (0.59–1.23) | (0.79–1.01) |
| **Ti** | 1.185 | (0.58–2.40) | (0.96–1.55) | **Ti** | 0.681 | (0.33–1.31) | (0.59–0.75) |
| **TIV** | 0.776 | (0.49–1.19) | (0.68–0.86) | **TIV** | 1.508 | (0.88–2.99) | (1.23–2.12) |
| **RR** | 0.771 | (0.22–1.29) | (0.55–0.92) | **RR** | 2.193 | (1.00–3.57) | (1.78–2.72) |
| **TIV_MIN_** | 0.591 | (0.17–1.22) | (0.39–0.80) | **TIV_MIN_** | 3.236 | (1.33–6.83) | (2.49–4.70) |
| **EELI** | 0.983 | (0.94–1.05) | (0.97–1.00) | **EELI** | 1.041 | (0.94–1.14) | (1.00–1.07) |

Supplementary table S3: Electrical impedance tomography (EIT) derived flow variables following etorphine and naltrexone administration, expressed as post-treatment-to-baseline ratios.
Data are presented as ratios calculated as the median value during the 10-minute period after drug injection divided by the corresponding baseline median value (baseline is represented by the pooled median of the measurements obtained from the 5-minutes before drug injection). A ratio of 1 indicates no change from baseline; values >1 indicate an increase, and values <1 indicate a decrease relative to baseline. For each variable, the table reports the group median ratio, range (minimum–maximum), and interquartile range (IQR) of the ratio.
Global inspiratory flow = PIF; global expiratory flow =PEF; right regional inspiratory flow = PIFR; right regional expiratory flow = PEFR; left regional inspiratory flow = PIFL; left regional expiratory flow = PEFL.
All EIT derived flow variables are expressed as normalized to tidal impedance variation (TIV).

| **Etorphine** | **Ratio** | **Range** | **IQR** | **Naltrexone** | **Ratio** | **Range** | **IQR** |
| --- | --- | --- | --- | --- | --- | --- | --- |
| **PIF/TIV** | 0.956 | (0.72–1.71) | (0.85–1.14) | **PIF/TIV** | 1.275 | (0.72–2.46) | (1.03–1.81) |
| **PEF/TIV** | 0.878 | (0.41–1.58) | (0.70–1.00) | **PEF/TIV** | 2.186 | (0.97–3.07) | (1.77–2.37) |
| **PIFR/TIV** | 0.884 | (0.63–1.55) | (0.76–1.09) | **PIFR/TIV** | 1.316 | (0.75–2.41) | (1.15–1.75) |
| **PEFR/TIV** | 0.753 | (0.45–1.61) | (0.65–0.94) | **PEFR/TIV** | 2.017 | (1.05–3.43) | (1.68–2.67) |
| **PIFL/TIV** | 0.958 | (0.69–1.60) | (0.84–1.18) | **PIFL/TIV** | 1.182 | (0.68–2.37) | (0.98–1.57) |
| **PEFL/TIV** | 0.874 | (0.45–1.70) | (0.71–1.03) | **PEFL/TIV** | 1.914 | (0.63–2.84) | (1.46–2.37) |
